# Microbial Transformation of Dietary Glycerol Contributes to Intestinal Acrolein Formation and Urinary Excretion

**DOI:** 10.1002/mnfr.70289

**Published:** 2025-10-29

**Authors:** Clarissa Schwab, Hanna Lang, Simone Stegmüller, Jiri Hosek, Angeliki Marietou, Lucia Huertas‐Díaz, Qing Li, Asta Petrine Smedgaard Krings, Andrea Zander, Ulrik Kræmer Sundekilde, Elke Richling

**Affiliations:** ^1^ Department of Biological and Chemical Engineering Aarhus University Aarhus Denmark; ^2^ Division of Food Chemistry and Toxicology Department of Chemistry RPTU University Kaiserslautern‐Landau Kaiserslautern Germany; ^3^ Technical University of Denmark Copenhagen Denmark; ^4^ Department of Food Science Aarhus University Aarhus Denmark

**Keywords:** 3‐HPMA, CEMA, diet, glycerol, gut microbiota

## Abstract

The aldehyde acrolein has been associated with diabetes, cardiovascular, respiratory, and neurodegenerative diseases, and gut microbiota possesses the potential for acrolein release via the key enzyme glycerol/diol dehydratase (PduCDE). This study aimed at estimating the contribution of gut microbiota to endogenous acrolein production. To minimize confounding sources, we investigated the intestinal acrolein‐producing potential of 20 volunteers housed under defined conditions. Glycerol was present in every meal and was detected in feces, suggesting availability to intestinal microbiota. Based on fecal metagenomics and *pduC* analysis, all volunteers showed potential for intestinal glycerol transformation to acrolein; the genus *Anaerobutyricum* was the major contributor across donors and time. Levels of urine biomarkers *N*‐acetyl‐*S*‐(3‐hydroxypropyl)‐L‐cysteine (3‐HPMA) and *N*‐acetyl‐*S*‐(carboxyethyl)‐L‐cysteine (CEMA) were higher after the consumption of meals with high glycerol levels, suggesting immediate microbial transformation to acrolein. Only a small proportion of acrolein metabolites was recovered in urine, possibly due to high compound reactivity. Donors could be separated into 3‐HPMA or CEMA phenotypes based on the predominance of urine biomarkers, and phenotypes related to overall fecal microbiota and fermentation metabolite profiles. Our data show that oral fat/glycerol intake together with intestinal microbiota activity might temporarily increase endogenous acrolein formation and that urinary biomarkers link to the intestinal microbiome.

## Graphical abstract

The double unsaturated aldehyde acrolein has been implicated in a number of human disorders. Investigating a cohort of 20 participants housed at defined conditions based on a combination of urinary and fecal biomarkers, we observed that glycerol was readily available to intestinal microbiota. All volunteers showed potential for intestinal microbial glycerol transformation to acrolein, and levels of urinary biomarkers were higher after the consumption of meals with high glycerol content, suggesting immediate intestinal glycerol transformation. Donors could be separated into different urinary biomarker phenotypes, and phenotypes related to overall fecal microbiota and fermentation metabolite profiles suggest a link between gut microbiota, liver, and kidney. The graphical abstract wass prepared using Biorender (License Nr. GS28VGTLZW).

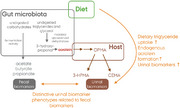

## 1 Introduction

The double‐unsaturated aldehyde acrolein is a highly reactive toxicant with the capacity to change the cellular redox potential and interact with DNA and proteins of eukaryotic and bacterial cells [1, 2, 3]. Acrolein has been associated with a number of health complications, including respiratory disorders (pulmonary edema, increased bronchial responsiveness, chronic and obstructive pulmonary disease), atherosclerosis and increased risk of cardiovascular disease, and neurodegenerative diseases such as Alzheimer's disease and multiple sclerosis [4, 5, 6]. Acrolein can enter the body through the respiratory system from the environment (combustion processes, smoking tobacco) or orally through diet [1]. Uptake has been linked to type 1 and 2 diabetes [7], and protein adducts have been related to diabetic nephropathy and retinopathy [8, 9]. Fried food, fruits, and fermented food were identified as dietary sources [10]. Furthermore, acrolein is endogenously generated during lipid peroxidation, amine oxidase‐mediated metabolism of polyamines, and myeloperoxidase activity [1].

**FIGURE 1:**
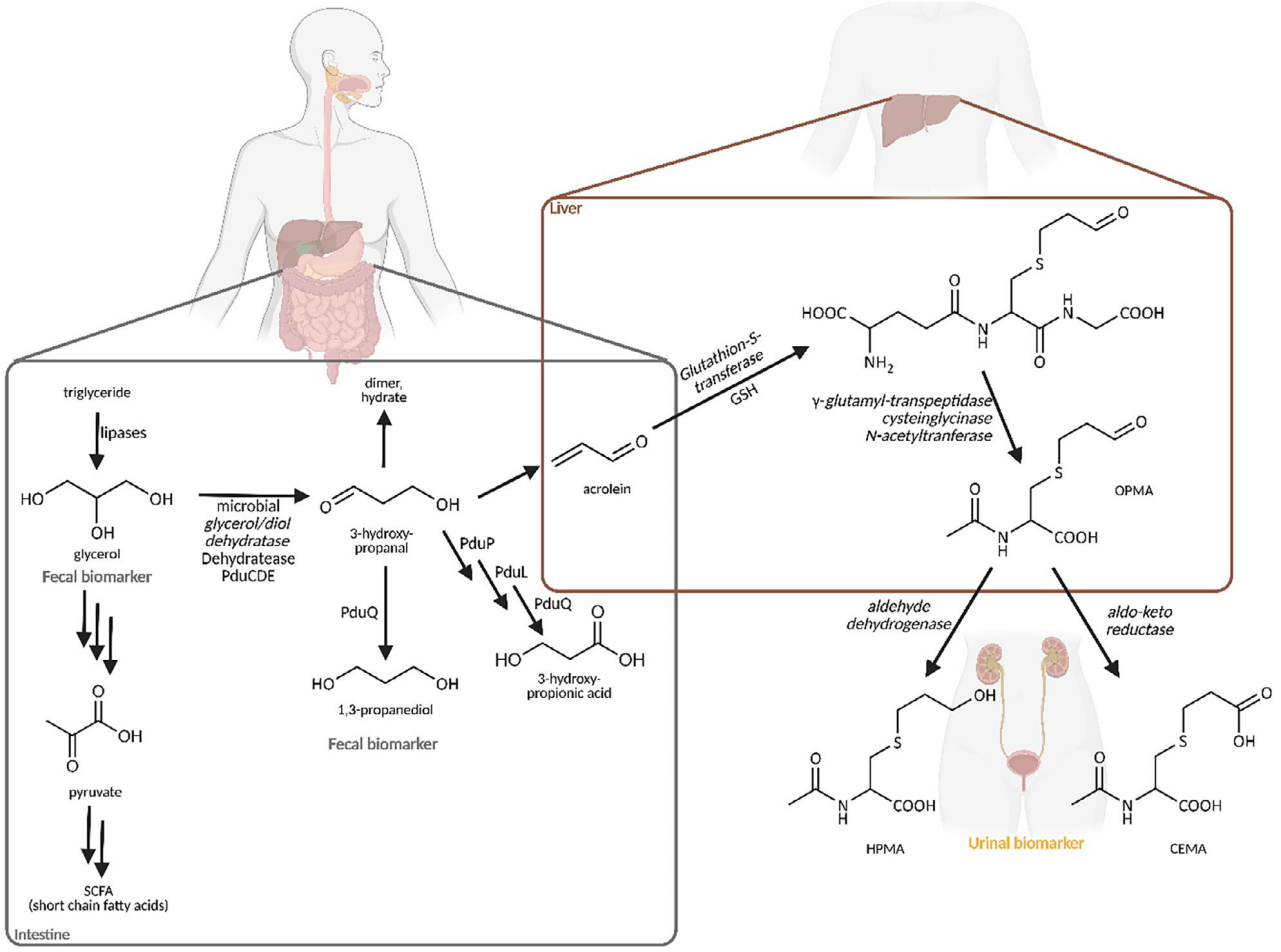
Microbial glycerol utilization and further host metabolism of acrolein. The figure has been generated using BioRender (Agreement Nr. YT28ULISMS).

In 2018, we suggested that intestinal microbial glycerol transformation should be considered an endogenous acrolein source [11] based on in vitro studies indicating glycerol metabolism/acrolein release by individual gut microbes, including *Anaerobutyricum hallii* and *Limosilactobacillus reuteri* [12, 13]. Microbial glycerol metabolism is catalyzed by enzymes/proteins encoded by the *pdu*‐*cbi*‐*cob*‐*hem* operon. The key enzymes are cobalamin‐dependent glycerol/diol dehydratases (PduCDE) (Figure 1), which convert glycerol to 3‐hydroxypropanal (3‐HPA). 3‐HPA can undergo further conversion to 1,3‐propanediol (1,3‐PD) and 3‐hydroxypropionic acid (3‐HP) or spontaneously dehydrate to acrolein at physiological conditions (Figure 1) [3]. Acrolein can also conjugate with dietary carcinogenic heterocyclic aromatic amines (HCA) to reduce their mutagenicity [12, 14], thus concurrently conferring beneficial and adverse bioactivities. In addition, *pdu* has a second substrate, 1,2‐propanediol (1,2‐PD), which is further metabolized to propanol and propionate with propanal as an intermediate [15, 16]. In vivo, 1,2‐PD metabolism contributes to the formation of the short‐chain fatty acid (SCFA) propionate [17].

The main route of acrolein elimination involves the conjunction with glutathione (GSH) in the liver and further transformation to *S*‐(3‐oxopropyl)‐*N*‐acetylcysteine (OPMA). OMPA is metabolized by aldo‐keto‐reductases to *N*‐acetyl‐*S*‐(3‐hydroxypropyl)‐L‐cysteine (3‐HPMA) or oxidized by the aldehyde dehydrogenase to *N*‐acetyl‐*S*‐(carboxyethyl)‐L‐cysteine (CEMA). An initiative to harmonize abbreviations suggested renaming 3‐HPMA to 3‐HPMA and CEMA to CoEMA [18]. 3‐HPMA is considered the primary metabolite of acrolein found in urine [19, 20] (Figure 1). Base levels and short‐term acrolein exposure can be tested noninvasively through the analysis of concentrations of 3‐HPMA and CEMA in collected urine [21, 22]. Based on urinary levels of these acrolein‐related biomarkers, Ruenz et al. [22] observed a substantial background of acrolein in nonsmoking volunteers living under defined conditions and diet, reinforcing again the idea of the intestinal microbiota as a substantial producer of endogenous acrolein.

**FIGURE 2:**
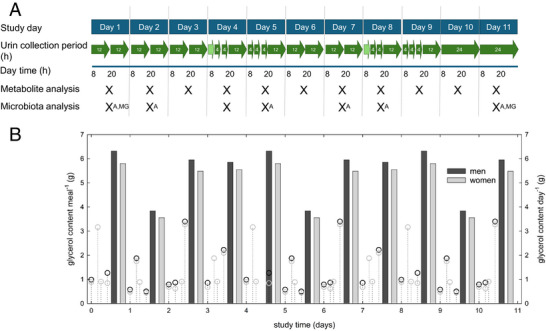
Study organization, sample collection, and estimated dietary glycerol intake. The study was run for 11 days starting at 8 am on the first day. All participants were provided with the same standardized diet. Urine and fecal samples were collected at regular intervals. (A) Urine was collected in specific time periods that ranged from 1 to 24 h. Fecal samples were collected once daily if available; fecal SCFA were analyzed from each sample. Samples collected on Days 1, 2, 4, 5, 7, 9, and 11 were used for microbiota analysis. ^A^Samples were analyzed using 16S rRNA gene amplicon sequencing; ^MG^Samples were used for metagenome sequencing unless indicated otherwise. (B) Estimated dietary glycerol content for men and women. Dots and glycerol levels are provided for each meal, and bars show glycerol levels per day.

Given the implication of acrolein in a broad range of health disorders, there is an urgent need to provide fundamental knowledge on the contribution of intestinal microbiota to endogenous acrolein formation. Therefore, the aim of our study was to investigate the relationship between diet and the fecal and urine metabolite biomarkers of glycerol metabolism (Figure 2A). Intestinal microbial activity leading to acrolein formation is difficult to quantify because of the high reactivity of the molecule, the host detoxifying mechanism, and the possibility for acrolein uptake from the environment [1]. To control for the impact of acrolein exposure from environmental and dietary sources, we investigated the glycerol‐dependent relationship of diet and gut microbiota in a cohort that was housed 11 days under defined conditions, as previously described [22, 23]. The study was originally conducted to evaluate the urinary excretion of biomarkers of furan and 2‐methylfuran (2‐MF) after the intake of coffee [23], and participants obtained a diet omitting fried and baked food items to prevent/reduce furan and also acrolein uptake through food items. We collected fecal samples and determined composition and functional potential for glycerol transformation with 16S rRNA gene sequencing, metagenomics, and quantitative PCR (qPCR) targeting *pduC* as a gene biomarker. Acrolein biomarkers 3‐HPMA and CEMA were analyzed in urine samples.

## 2 Experimental Section

### 2.1 Ethical Approval and Study Cohort

The study entitled “Dosimetry of human exposure to furan and 2‐methylfuran by monitoring urinary biomarkers” was performed in 2021 [23]. The study was approved by the Ethics Commission in Mainz, Germany, with amendment (study no. 2018–13870_1 and 2018–13780_2). The study was an 11‐day diet human intervention study in a controlled environment with a controlled diet (Table S2) that has been described in detail [23]. Briefly, the study was performed with 20 nonsmoking male (*n* = 10) and female (*n* = 10) volunteers under tightly controlled environmental and nutritional living conditions. All volunteer participants completed the study.

### 2.2 Diet and Calculation of Glycerol Content

During the study, participants consumed the same standardized omnivorous diet containing boiled meat and vegetables, fruits, and dairy products. Diets consisted of unheated or mildly heated foods (≤ 100°C) such as yogurt, fruits, vegetables, boiled meat, potatoes, noodles, and rice. Each participant was provided with four meals each day, and there were four repetitive diet schedules (Days 1, 5, and 9; Days 2, 6, and 10; Days 3, 7, and 11; and Days 4 and 8, Table S1). The total kcal intake of women (≈ 2143 kcal/day) was about 300 kcal less than that of men (≈ 2430 kcal/day) (Figure S1). Carbohydrates were the main dietary macromolecules (Figur1A,B). Water was allowed ad libitum.

Based on fat content in the individual food items, the content of glycerol was calculated using (i) the assumption that the major part of fat is constituted by triglycerides [24] and (ii) the amount of glycerol can be calculated with a correction factor accounting for different lengths of fatty acids of the food items [25].

### 2.3 Sample Collection

All participants provided urine samples (10 male, 10 female), and *n* = 19 participants (10 male, 9 female) agreed to donate fecal samples. Urine samples were collected over a period of time as indicated in [23] (Figure 2A). The total urine volume was calculated using the urine weight and assuming a density of 1 kg L^−1^. Fecal samples were collected by the volunteers once a day, if available. Donors donated between 3 and 11 samples during the intervention, with a median of *n* = 9. Collected urine and fecal samples were stored at −20°C prior to analysis. Fecal samples were shipped on dry ice to Aarhus University shortly after the study for further processing.

### 2.4 DNA Isolation

DNA from fecal samples was isolated using the FastDNA Spin Kit for Soil (MP Biomedicals) following instructions. In total, 15, 14, 19, 13, 16, 17, and 14 samples were available on Days 1, 2, 4, 5, 7, 8, and 11, respectively. Samples were lysed at 6.0 m s^−1^ for 40 s using Lysing Matrix E tubes and a FastPrep‐24 instrument (MP Biomedicals). DNA was eluted with nuclease‐free water. The quality of DNA was evaluated by agarose gel electrophoresis to test DNA degradation, and the concentration of DNA was measured with Nanodrop.

### 2.5 Microbiota Profiling With 16S rRNA Gene Sequencing and Data Analysis

For library preparation, a two‐step PCR approach was used according to the 16S Metagenomic Sequencing Library Preparation guide of Illumina. Briefly, the V3‐V4 hypervariable region of the 16S rRNA gene was amplified using Bac341F and Bac805R with adapters and libraries were further prepared as described [26]. Sequenced reads were further processed as outlined [26] combining cutadapt for primer removal and the dada2 package for quality filtering and read merging. Amplicon sequence variants (ASV) were taxonomically annotated using the IDTAXA classifier with the Silva v138 database. The median number of reads per processed sample was 14 744 reads (range 10 388–24 038 reads), and the negative control yielded 30 reads.

### 2.6 Metagenomic Sequencing and Data Processing

We generated metagenomes of *n* = 2 fecal samples of each participant. Samples were preferably collected at the beginning (Days 1 or 2) and the end (Days 10 and 11) of the study; from one participant, fecal microbiota was only sequenced once (total number of samples, *n* = 37). DNA samples were sent for shotgun sequencing using Illumina NextSeq (Novogene Europe). BBMap (v.38.71) was used to quality control reads from all samples by removing adapters, reads that mapped to quality control sequences (PhiX genome), low‐quality reads (*trimq = 14, maq = 20, maxns = 1, and minlength = 45*), and host reads (human genome). Quality‐controlled reads were assembled into scaffolded contigs using the metaSPAdes (v3.15.5) [27]. Scaffolds were length‐filtered (≥ 1000 bp), and quality‐controlled reads from each metagenomic sample were mapped against the scaffolds of each sample. Mapping was performed using BWA (v0.7.17‐r1188; *‐a*) [28]. BAM files were processed using the *jgi_summarize_bam_contig_depths* script of MetaBAT2 (v2.15) to compute within‐ and between‐sample coverages for each scaffold [29]. The scaffolds were binned by running MetaBAT2 on all samples individually (*–minContig 2000 and –maxEdges 500*). Metagenomic bins were quality‐controlled using the CheckM (v1.2.3) [30] lineage workflow (completeness ≥ 50% and contamination < 10%) to generate metagenomic assembled genomes (MAGs). Quality‐controlled MAGs were annotated using Bakta (v1.10.4) [31], and MAGs were taxonomically annotated with GTDBtk (v2.4.0) [32]. A representative set of MAGs (rMAGs) was generated by clustering all MAGs using the dRep (v3.5.0, *S_ani = 0.95*) [33] dereplicate workflow. Quality‐controlled reads were also taxonomically classified using Sylph with default settings [34].

### 2.7 qPCR

qPCR was conducted to quantify selected bacterial taxa with *pduC* detected using MAG analysis in this and a previous study, that is, *A. hallii* and *A. soehngenii, Mediterraneibacter* (formerly *Ruminococcus*) *gnavus, Blautia obeum*, and *Flavonifractor plautii*, in addition to *Clostridium* senso stricto, which also harbour *pdu* [35, 36]. Abundance was quantified in fecal samples collected on Days 1, 2, 4, 5, 7, 8, and 11 (Table S2). Standards were prepared, and qPCR was run as previously described [35]. Briefly, a tenfold dilution series of each linearized plasmid containing the target gene was included in the run to determine the linear range and the limits of detection (Table S2). A two‐step PCR protocol was run for 40 cycles, followed by melting curve analysis. Each run contained negative controls without template DNA.

### 2.8 Substrate and Metabolite Analysis Using High‐Performance Liquid Chromatography With a Refractive Index Detector (HPLC‐RI)

A 1260 Infinity II LC system with RI detector was used to determine metabolites in all available fecal samples, including the major SCFAs acetate, propionate, and butyrate, and the glycerol transformation product 1,3‐PD as described [35]. Metabolites were extracted from 200 to 300 mg of feces with 5 mM H_2_SO_4_ and were analyzed directly using a Hi‐Plex‐H column connected to a guard column. The mobile phase was 5 mM H_2_SO_4_ with a flow rate of 0.6 mL min^−1^ at 40°C. External standards were used for quantification, and the minimum detection limit was 0.01 mM.

### 2.9 ^1^H‐Nuclear Magnetic Resonance Spectroscopy (^1^H‐NMR)

We used ^1^H‐NMR spectrometry to verify the presence of glycerol in feces, as the peak of glycerol had a similar retention time as lactate and 3‐HP in the HPLC‐RI chromatograms. Glycerol and 1,3‐PD were analyzed from all collected fecal samples as described [37]. Briefly, 400 µL of the cell‐free supernatants were mixed with 200 µL D_2_O and 0.32 mM trimethylsilylpropanoic acid (TSP), and the mixture was transferred into 5‐mm NMR tubes. ^1^H‐NMR spectroscopy was performed using a Bruker NEO‐IVDR 600 NMR spectrometer, operating at a ^1^H frequency of 600.03 MHz and with a 5‐mm ^1^H BBI probe (Bruker BioSpin). TSP was used as an internal chemical shift reference and quantification standard, and all ^1^H spectra were referenced to the TSP signal at 0 ppm. The manual phase and baseline spectra were corrected using Topspin 4.09 (Bruker BioSpin). Signals were assigned and quantified using Chenomx NMR Suite 10.1 (Chenomx Inc.).

### 2.10 LC‐MS/MS Analysis to Determine Urinary Levels of Acrolein Biomarkers

Urine samples were prepared and analyzed as described before [22] without solid‐phase extraction. In brief, samples were diluted if necessary, acidified with acetic acid (pH 2.5 ± 0.5), and internal standards (50 ng mL^−1^ D_3_‐CEMA and 150 ng mL^−1^ D_3_‐HPMA) were added. Before the measurement, samples were membrane filtered. The calibration curves for 3‐HPMA and CEMA were in the range of 0.5 to 100 ng mL^−1^. The column was an Agilent Zorbax Eclipse XDB‐C18 (50 × 4.6 mm, 1.8 µm), with the mobile phases (A) 0.1% acetic acid in water and (B) MeCN, with a flow of 0.6 mL min^−1^. The gradient program was as follows: 0.0–2.0 min, 1% B; 2.0–5.0 min, increase to 10% B; 5.0–5.1 min, increase to 90% B, 5.1–8.1 min, 90% B; 8.1–8.2 min, reduction to 1% B; 8.2–12.0 min, 1% B. The injection volume was 2 µL. The expected retention times of 3‐HPMA and CEMA were 5.4 and 5.6 min, respectively. Signals were detected in ESI^−^_,_ sMRM mode.

### 2.11 Statistics

The normality of the data was tested using the Shapiro–Wilk test. Depending on normality, one way ANOVA or the nonparametric methods, Kruskal–Wallis test with the Tukey post hoc test, or the Mann–Whitney *U* rank‐sum test, implemented in SigmaPlot v14 was used to test α‐diversity indices or qPCR data. Statistical analysis of 16S rRNA gene amplicon libraries, including PERMANOVA, was conducted with QIIME 2 version 2023.9.1 [38]. Alpha‐diversity metrics (observed features and Chao and Shannon index) were estimated using q2‐diversity after samples were rarefied (subsampled without replacement) to 10 388 sequences per sample. The rarefied data was also used for β‐diversity calculation (Bray Curtis) in the Python library scikit‐bio. Correlation analysis between the relative abundance of the 20 most abundant genera and major fecal SCFA was performed with the “rstatix” package in R using the Spearman correlation test. For linear discriminant analysis (LDA), we used the package MicrobiotaProcess [39] with the following setting: an initial Kruskal–Wallis test with a *p* value filtration of 0.05 and a second Wilcox test with a *p* value filtration of 0.01. Only LDA scores above 2 were considered significant.

### 2.12 Data Availability 

16S rRNA gene data and metagenome sequences are available at the European Nucleotide Archive (ENA) under accession number PRJEB90537.

## 3 Results and Discussion

### 3.1 Glycerol Was Provided With Every Meal, With Levels Varying Between Meals and Days

As we were mainly interested in the fate of glycerol, we calculated the dietary glycerol content with the assumption that triglycerides were the major fat constituents [24]. Based on our estimate, daily glycerol intake ranged from 3.8 to 6.3 and 3.6 to 5.8 g day^−1^ for men and women, respectively (Figure 2B). On Days 1, 2, 5, 6, 9, and 10, glycerol uptake was highest with lunch (49–55%), while on Days 3, 4, 7, and 8, a major proportion of glycerol (38–60%) was provided with dinner (Figure 2B). These data indicate that glycerol was provided with every meal, with variations due to the different diet schemes.

### 3.2 Participants Maintained Individual Fecal Microbiota Profiles While Consuming the Standardized Diet

**FIGURE 3:**
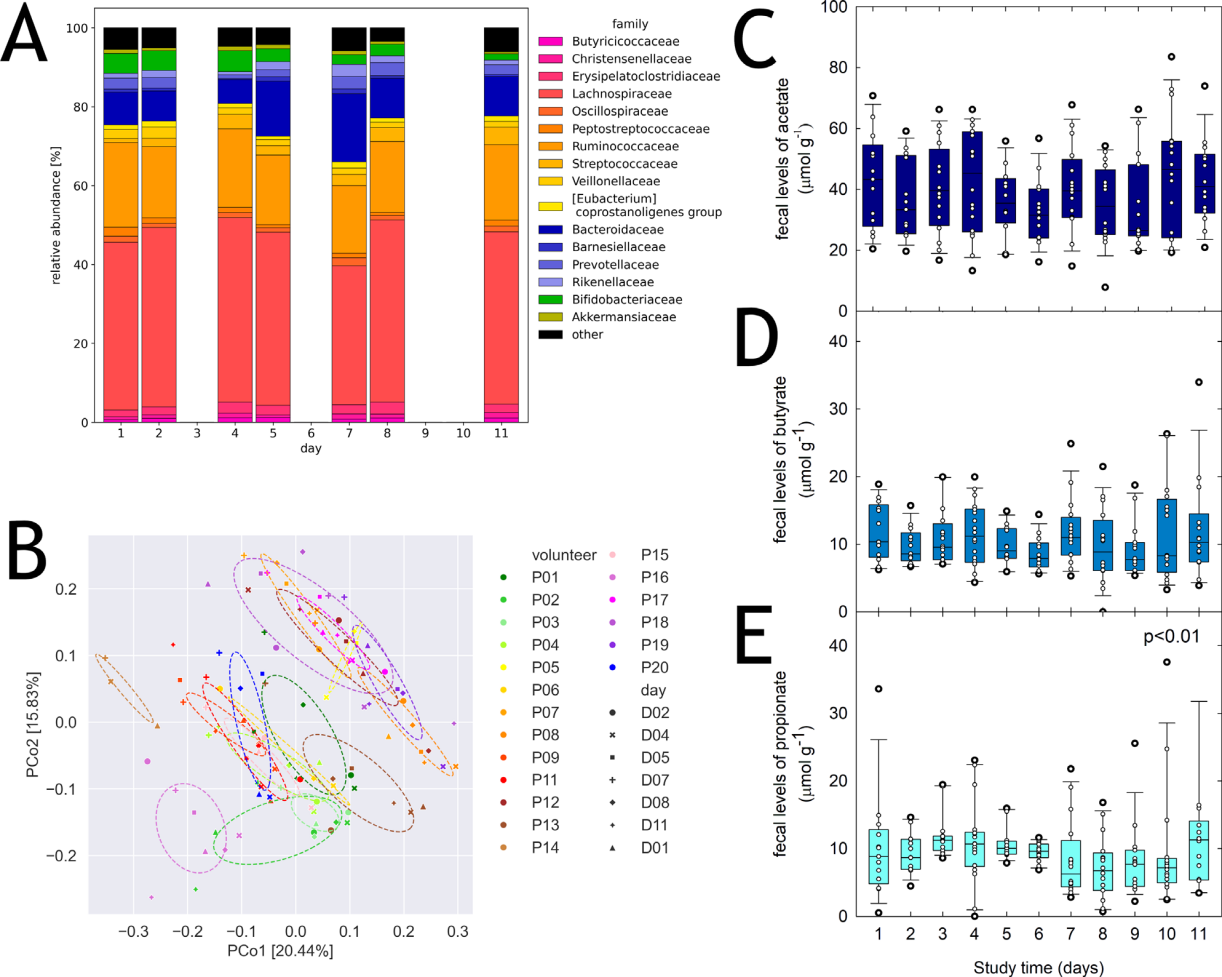
Fecal microbiota composition and SCFA profiles. Microbiota composition was determined using 16S rRNA gene amplicon sequencing, and levels of fecal fermentation metabolites acetate, butyrate, and propionate were analysed with HPLC‐RI. (A) Relative abundance of major bacterial families. (B) Beta‐diversity analysis based on Bray‐Curtis and clustered by participants. (C) Fecal acetate levels at each study day. (D) Fecal propionate levels at each study day. (E) Fecal butyrate levels at each study day. (C)–(E) Shown are boxplots with median and 25th and 75th percentiles; whiskers indicate the 5th and 95th percentiles, and each individual sample is presented as a white circle. Statistical difference was determined using the Kruskal–Wallis test with all pairwise multiple comparison procedures (Tukey test); *p* < 0.05 was considered significant. SCFA, short‐chain fatty acid.

To determine the impact of the standardized diet on fecal microbiota composition and fermentation profiles, we conducted 16S rRNA gene amplicon sequencing and monitored levels of major SCFA acetate, propionate, and butyrate with HPLC‐RI. The major bacteria families were *Lachnospiraceae, Ruminococcaceae*, and *Bacteroidaceae* (Figure 3A). Based on α‐diversity indices Chao and Shannon, there were no significant changes in observed richness or evenness across days but between participants (Figure S2). Beta‐diversity based on Bray‐Curtis was influenced by the donor microbiota (PERMANOVA, *p* < 0.001) and not by study day (Figure 3B). Acetate was the major fecal SCFA, contributing a median of 68.6% to total SCFA (sum of acetate, propionate, and butyrate) (interquartile range 25% (IQR25): 62.7%, IQR75 76.6%), followed by butyrate (median 18.4%, IQR25: 16.1%, IQR75: 20.7%) and propionate (median 8.6%, IQR25: 6.3%, IQR75: 18.9%) (Figure 3C–E). There was a difference of fecal propionate levels between days (*p* < 0.01). We selected the 20 most abundant genera and correlated relative abundance to SCFA profiles (Figure S3). Relative abundance of *Dorea, Anaerostipes*, unclassified *Lachnospiraceae*, and *A. hallii* was positively related with propionate levels (*p <* 0.05) in agreement with the ability of *Anaerobutyricum* sp. to metabolize 1,2‐PD [15]. Taken together, our results show a high impact of donor individuality on fecal microbiota composition, which was not majorly affected by short‐term diet standardization in agreement with previous investigations [40].

### 3.3 Fecal Microbiota Had the Potential to Metabolize Glycerol via Pdu

Next, we identified the potential of the fecal microbiota of the study participants for glycerol transformation using shotgun metagenome sequencing as an untargeted approach. In total, 3955 MAGs were constructed, and *n* = 2512 MAGs passed the criteria of completeness. MAGs were grouped in *n* = 399 species‐level clusters (representative MAGs, rMAGs). rMAGs were annotated to eight phyla of the domain *Bacteria*, and to *Archaea* of the *Methanobacteria*. The majority of rMAGs were assigned to *Bacillota* (73.4%), followed by *Bacteroidata* (13.5%), *Actinomycetota* (7.8%), and *Pseudomonadota* (3.5%). Results were in agreement with the taxonomic distribution of metagenomes based on sylph (Figure S4) and with profiling based on 16S rRNA gene amplicon sequences (Figure 3A).

**FIGURE 4:**
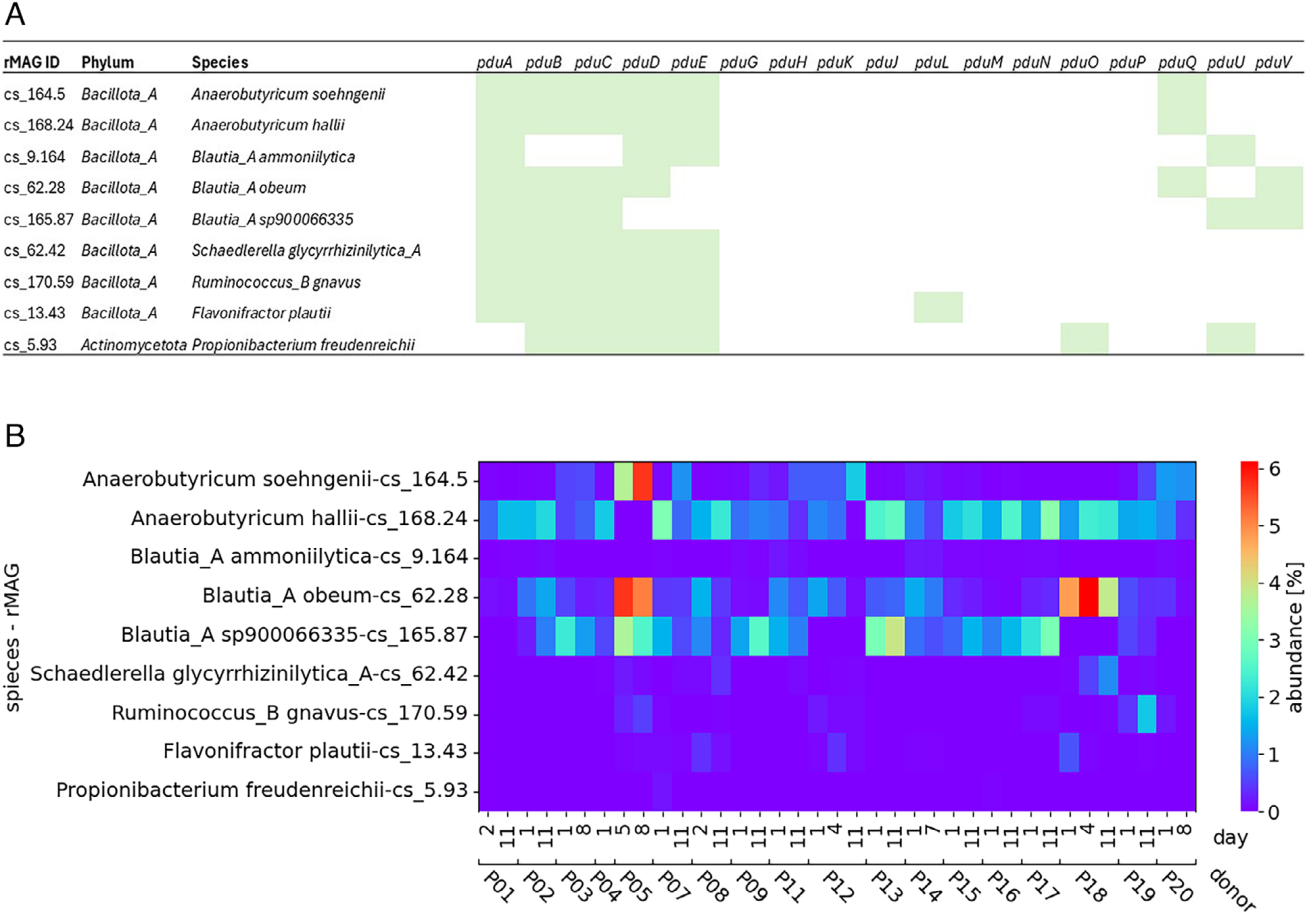
Taxa contributing *pdu* and occurrence in feces. rMAGs that contained *pduC* were identified (*n* = 9) using output from Bakta annotations, and the presence or absence of individual genes of the *pdu* loci was determined. (A) Presence/absence of genes associated with *pdu* in nine *pduC*‐harboring rMAGs. (B) Relative abundance of rMAGs with *pduC* in metagenomes from individual donors as determined by sylph profiler.

To obtain taxa with the potential to metabolize glycerol and 1,2‐PD, we identified rMAGs that harbored *pduCDE* and/or other key genes of the *pdu* operon. In total, nine rMAGs (2.2% of all rMAGs) possessed at least one subunit encoding gene of *pduCDE* (Figure 4A). The majority (*n* = 7) of rMAGs harboring *pduC*, *pduD*, and/or *pduE* were assigned to *Lachnospiraceae*, one to *Oscillospiraceae*, and one to *Propionibacteriaceae*. The predominant species included previously identified glycerol transformers *A. soehngenii*, *A. hallii*, *B. obeum, M. gnavus, F. plautii*, and *Propionibacterium freudenreichii* [15, 16, 41] in addition to *Blautia ammoniilytica*, *Blautia sp900066335*, and *Mediterraneibacter glycyrrhizinilytica*. Based on re‐identification of rMAGs harboring *pdu* in individual donor samples (Figure 4B), *A. hallii* and *A. soehngenii* were present in 95% and 89% of samples at a relative abundance of 1.39% and 0.09%. *B. obeum* (0.46%)*, B. ammoniilytica* (0.02%), and *B. sp900066335* (0.88%) were detected in 100%, 76%, and 73%. *M. gnavus* and *M. glycyrrhizinilytica* (both 0.01%) were detected in 68% and 57%, while *Flavonifractor* and *Propionibacterium* were present in 34 and 6 out of 37 samples at median abundance levels ranging from 0.02% to 0% (Figure 4B). Each donor harbored at least five species potentially contributing to Pdu activity, with a median of 7. *Anaerobutyricum, Blautia*, and *Medierraneibacter* spp. are considered common gut commensals. In contrast, *P. freudenreichii* is a food culture that is used as starter in Swiss Emmental‐type cheeses [41]. *P. freudenreichii* was only detected in the feces of six participants at the beginning of the study, suggesting that it was present as a transient (allochthonous) culture with an origin in fermented food.

**FIGURE 5:**
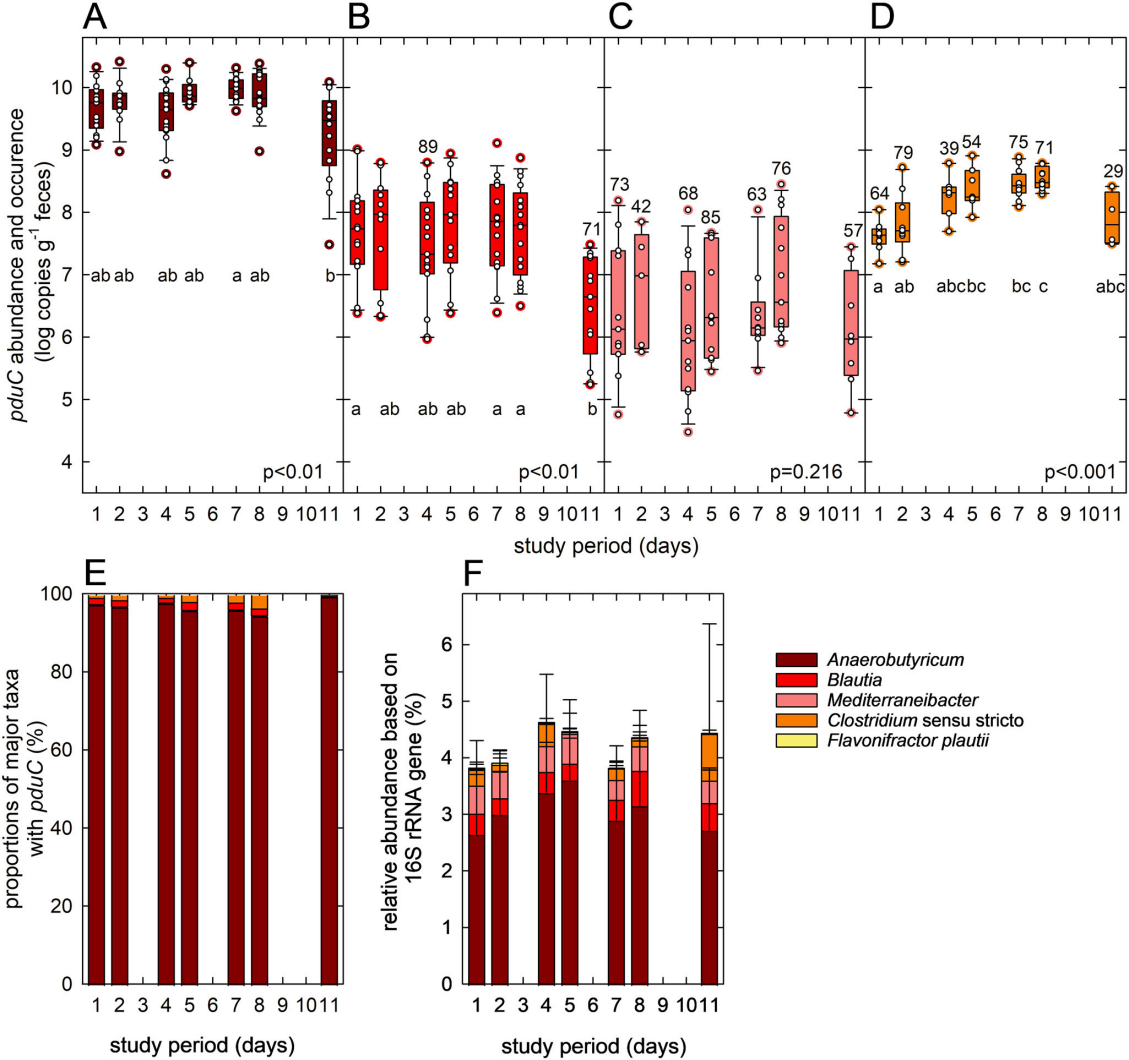
Abundance and occurrence of fecal gene biomarker *pduC*. Abundance of major taxa contributing *pduC* was determined by qPCR (A–E) and 16S rRNA gene amplicon sequencing (F). (A) Abundance of *A. hallii* and *A. soehngenii*, (B) Abundance of *B. obeum*, (C) Abundance of *M. gnavus*, and (D) Abundance of *Clostridium sensu stricto*. If the occurrence was < 100%, the actual levels are indicated. (A)–(D) Shown are boxplots with the median and 25th and 75th percentiles; whiskers indicate the 5th and 95th percentiles, and each individual sample is presented as a white circle. Abundances were compared with the Kruskal–Wallis test with the Mann–Whitney *U* pairwise tests and Bonferroni‐corrected *p* values. (E) Proportions of major taxa contributing *pduC* based on qPCR data. (F) Relative abundance of *Anaerobutyricum, Blautia, Mediterraneibacter, Flavonifractor*, and *Clostridium sensu stricto* based on 16S rRNA gene sequencing.

During the study, *Anerobutyricum* was most abundant (log 7.5–10.1 cells g^−1^ feces) based on qPCR, contributing > 93% of all *pduC*, followed by *Clostridium* sensu stricto, *B. obeum*, and *M. gnavus* (Figure 5A–E). *pduC* of *Clostridium* sensu stricto was identified by qPCR but was not detected within the assembled rMAGs. *pduC* of *F. plautii* was only detected in 2 samples, in agreement with low abundance in metagenomes. On Day 11, the abundance of *Anaerobutyricum* and *Blautia* spp. was significantly lower than on Days 1 and 4, and cell counts of *Clostridium* sensu stricto were significantly higher on Days 5, 7 and 8 compared to Day 1 (Figure 5B,D) indicating shifts within the *pdu* community. In parallel, we monitored the relative abundance of our target taxa using 16S rRNA gene amplicon sequencing targeting the V4 region. In agreement with qPCR results, *Anaerobutyricum* was more abundant than the other taxa (Figure 5F).

In summary, our data based on fecal sample analysis, indicate that the intestinal microbiota of human adults possessed the steady potential for glycerol transformation via *pdu*. In agreement with previous studies, the ability for Pdu‐driven glycerol formation was shared by few taxa, and the human fecal *pdu*‐harboring community was dominated by *Anaerobutyricum* sp. [16, 42].

### 3.4 Glycerol Was Present in Feces, While the Glycerol Transformation Metabolite 1,3‐PD Was Not Detectable

**FIGURE 6:**
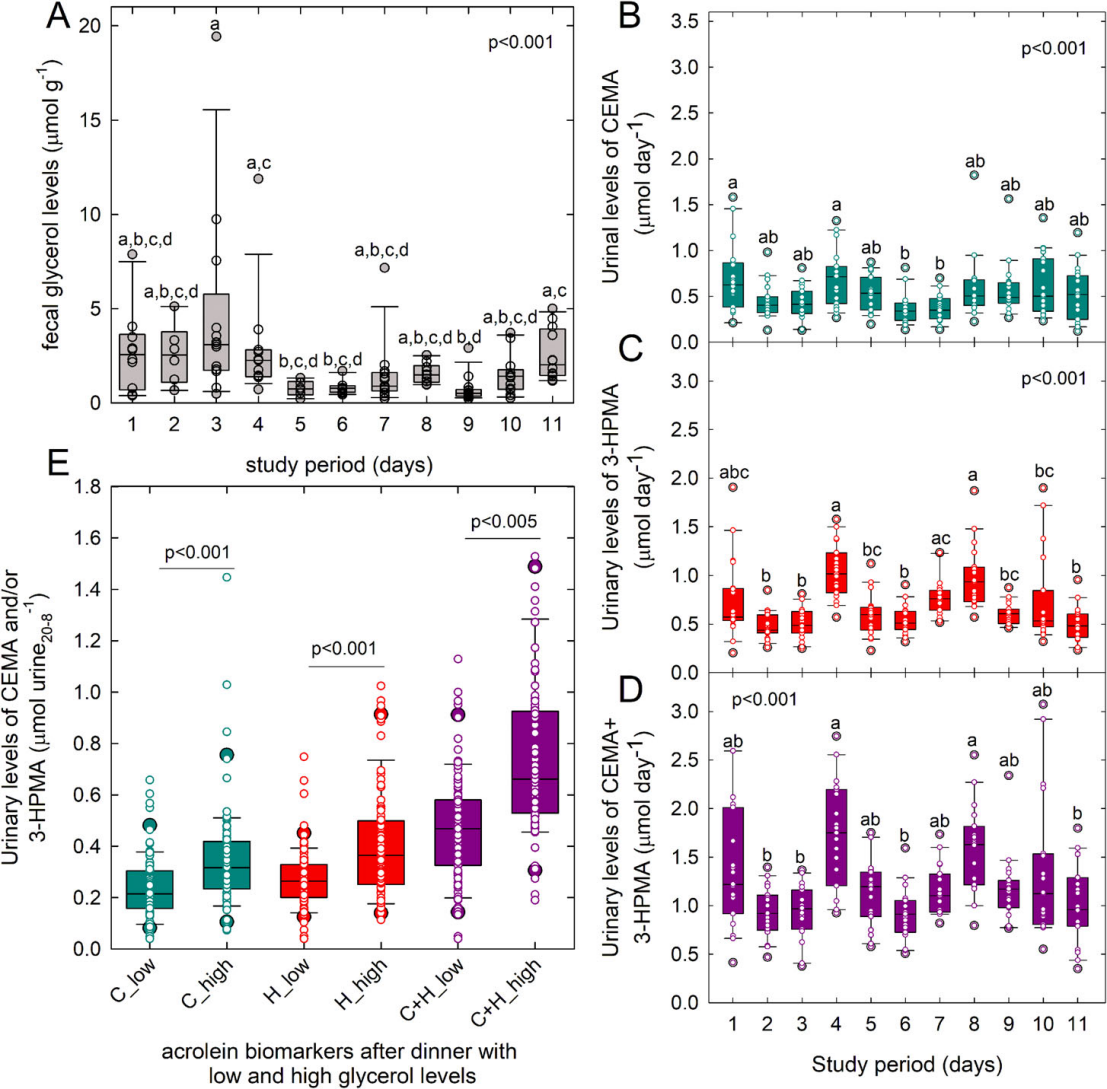
Levels of fecal glycerol and urinary glycerol/acrolein biomarkers. Glycerol was determined in feces using ^1^H‐NMR, while urine levels of CEMA and 3‐HPMA were determined with LC‐MS. (A) Fecal glycerol levels. (B–D) Urinary levels of CEMA (B), 3‐HPMA (C), and the sum of CEMA + 3‐HPMA (D). Differences in daily levels were determined using Kruskal–Wallis one way analysis of variance on ranks followed by Tukey test all pairwise multiple comparison procedures, *p* < 0.05%, was considered significant. (E) Urine levels of CEMA, 3‐HPMA, and CEMA + 3‐HPMA in urine collected overnight between 8 p.m. and 8 a.m. after dinner with low and high glycerol content. Differences in levels were determined using the Mann–Whitney *U* rank‐sum test and *p* < 0.05% was considered significant. (A)–(E) Shown are boxplots with median and 25th and 75th percentiles; whiskers indicate the 5th and 95th percentiles, and each individual sample is presented as a white circle.

^1^H‐NMR spectrometry was employed to track fecal glycerol levels since fecal microbiota analysis based on metagenome and *pduC* biomarkers suggested that all donors had the potential to intestinally transform glycerol. Glycerol was detected in all analyzed fecal samples, and levels ranged from 0.2 to 19.4 µmol g^−1^ feces (Figure 6A). Glycerol levels were significantly higher on Day 3 compared to Days 5 and 6 (Figure 6A). Median fecal glycerol levels of each participant ranged from 0.5 to 3.2 µmol g^−1^ feces but were not significantly different between participants (*p* = 0.252, Figure S5A).

While CEMA and 3‐HPMA are general indicators of host acrolein exposure [19, 20, 21, 22], 1,3‐PD is, to our best knowledge, exclusively derived from microbial glycerol transformation. Despite the detection of fecal glycerol suggesting availability for transformation by intestinal microbiota, 1,3‐PD was not detected at levels ≥ 0.2 µmol g^−1^ feces based on analysis with HPLC‐RI and ^1^H‐NMR. In vitro, accumulation of intermediates (3‐HPA/acrolein) and the formation of 1,3‐PD and 3‐HP differed between microbial species with PduCDE and between growth conditions. For example, the gut symbiont *L. reuteri*, which is usually low abundant in human feces [16, 42], metabolized some of the provided glycerol to 1,3‐PD, and the proportion of 1,3‐PD increased if glucose was available [43]. In contrast, strains of *A. hallii* and *A. soehngeniii* formed low amounts of 1,3‐PD (1 mM or less) when incubated with glycerol or glucose and glycerol [15]. Conversion of 3‐HPA to 1,3‐PD is catalyzed by 1,3‐propanediol oxidoreductase (PduQ), with NADH as a cofactor [15]. *L. reuteri* provides NADH through concurrent carbohydrate metabolism [43], while *A. hallii* seems to lack such allocation pathways. When *A. hallii* was added to intestinal microbiota in vitro, there was lower 1,3‐PD formation in some samples compared to incubations without supplementation of *A. hallii* [42], indicating the potential of *A. hallii* to drive glycerol transformation within complex microbial communities.

Taken together, these observations suggest that glycerol was available to the intestinal microbiota, but that 1,3‐PD was likely not produced because *Anaerobutyricum* was the predominant taxon with potential for PduCDE activity.

### 3.5 Urine Biomarkers Suggest Microbial Acrolein Release Depending on Dietary Glycerol Uptake

While 1,3‐PD might not be a major glycerol metabolite of *Anaerobutyricum*, members of this genus have been shown to effectively release acrolein from glycerol in single culture and within a complex microbial community [13, 42, 44]. To estimate intestinal acrolein formation, we determined the metabolite biomarkers CEMA and 3‐HPMA in the urine samples of all volunteers.

CEMA and 3‐HPMA were detected in all samples, and daily levels ranged from 0.11 to 1.82 µmol day^−1^ and 0.20 to 1.90 µmol day^−1^ for individual participants, respectively (Figure 6B,C). Median daily levels of extracted CEMA, 3‐HPMA, and CEMA+3‐HPMA were within the range of 0.34–0.71, 0.44–1.02 and 0.91–1.75 µmol day^−1^, respectively (Figure 5B–D). The sum of CEMA+3‐HPMA was significantly higher at Day 4 compared on Day 2, and on Day 8 compared to day 6 (Figure 6D), possibly due to coffee consumption on Days 4 and 8 at breakfast [23]. In previous studies, the presence of acrolein in roasted coffee beans depended on preparation procedures [45, 46], and in coffee, up to 0.81 µg mL^−1^ acrolein was detected [47]. With the assumption that similar levels were present in the coffee consumed in our study, 3.6 and 7.2 µmol acrolein could have been taken up at Days 4 and 8. To relate dietary glycerol intake (mainly via triacylglycerides) and urinary acrolein levels without the confounding factor of coffee consumption, we compared CEMA and 3‐HPMA levels in urine collected overnight between 20 p.m. and 8 a.m. following days with “high” glycerol levels at dinner (Days 3, 4, 7, 8) with days with “low” glycerol (Days 1, 2, 5, 6, 9) and omitted Days 10 and 11 when urine samples were collected over a period of 24 h (Figure 2A). In previous studies, meals with fried food that contained fat and acrolein (e.g., fried chicken, French fries, or potato crisps) led to an increase in urinary 3‐HPMA excretion within the first 2 h after consumption, with maximum levels after 8–12 h [21, 48]. Here, we observed that median urine levels of CEMA, 3‐HPMA, and the sum of CEMA + 3‐HPMA were significantly higher in urine collected 2–14 h after the consumption of dinner with “high” glycerol levels compared to meals with “low” glycerol (Figure 6E). In our study, meals were prepared to avoid the formation of heat‐induced contaminants such as furan and acrolein (e.g., boiling rather than frying, no overheating) [23]. Therefore, we have a strong indication that intestinal microbial transformation of dietary glycerol was the reason for higher levels of acrolein biomarkers.

### 3.6 Estimated Levels of Intestinal Acrolein

Based on rat studies that orally administered ^14^C‐labelled acrolein, about 20% of the provided total dose was excreted as 3‐HPMA [49]. Under the assumption that a similar ratio prevails in humans, intestinal levels of acrolein can be estimated at 1.32 and 1.83 µmol after “low” and “high” glycerol dinners with median 3‐HPMA levels of 0.26 and 0.33 µmol in urine collected between 2 p.m. and 8 a.m. overnight. Calculated glycerol levels in dinner meals ranged from 0.44 to 1.26 (“low”) to 2.1–3.4 g glycerol meal^−1^ (“high”). With an expected efficiency in lipid hydrolysis to glycerol and free fatty acids of 95% [24, 50], 0.24–0.68 and 1.13–1.84 mmol glycerol reached the colon, taking into account differences in glycerol level in meals prepared for male and female participants. Together, these data suggest that on days with “low” glycerol dinners, 0.03–0.10 mol% of glycerol was transformed to acrolein, while at days with “high” glycerol meals, 0.02–0.03 mol% was transformed, suggesting a nonlinear relationship of glycerol availability and further transformation to intestinal acrolein and urinary mercapturic acids. Intestinal microbes with *pdu* compete with other pathways for glycerol utilization: for example, when glycerol is metabolized to pyruvate [51], it can yield a variety of SCFA. In addition, acrolein is a highly reactive chemical. In a previous in vitro study that employed fecal microbiota batch fermentations, we estimated that between 1/6 and 1/200 of the provided glycerol (100 mM) was transformed by intestinal microbiota to acrolein. Free acrolein levels were low, and > 70% of acrolein was predicted to be in the bound state [42].

In summary, our observations indicate that in vivo, dietary glycerol led to higher excretion of acrolein biomarkers after intestinal microbial glycerol transformation. At the same time, the proportion of glycerol‐derived acrolein that was traceable as urinary biomarkers was low, possibly due to parallel microbial metabolic pathways and immediate binding of acrolein to cellular material.

### 3.7 Participants Could be Distinguished Based on Urine 3‐HPMA and CEMA Phenotypes That Related to *pduC* Profiles and Fecal SCFA

In humans, 3‐HPMA is often the main urinary mercapturic acid biomarker, and CEMA is present in lower levels [21, 22]. In agreement, the median ratio of CEMA/3‐HPMA per day was 0.73 in our study. A similar cohort with defined housing conditions and diet observed a ratio of 0.33, and studies investigating short sampling intervals after the consumption of potato crisps [21, 22] reported ratios of 0.63 to 1.67, indicating that dietary intake and/or sampling schemes could be contributing factors. Here, we provided a standardized diet, and most participants (14/20) had consistently higher levels of 3‐HPMA than CEMA in urine on most days of the intervention (Table S3). At the same time, we observed that for 6/20 participants, levels of 3‐HPMA were lower than CEMA at most study days, and/or there were higher median levels of CEMA than 3‐HPMA (Table S3), suggesting the presence of rather persistent 3‐HPMA and CEMA phenotypes of urinary excreted mercapturic acids linked to acrolein. The occurrence of 3‐HPMA and CEMA phenotypes could be due to polymorphisms or differences in regulation of aldo‐keto‐reductases and aldehyde dehydrogenases, which transform OPMA to 3‐HPMA and CEMA, respectively, similar to what is observed for glutathione‐*S*‐transferases that catalyze the conjugation of acrolein and glutathione [52].

**FIGURE 7:**
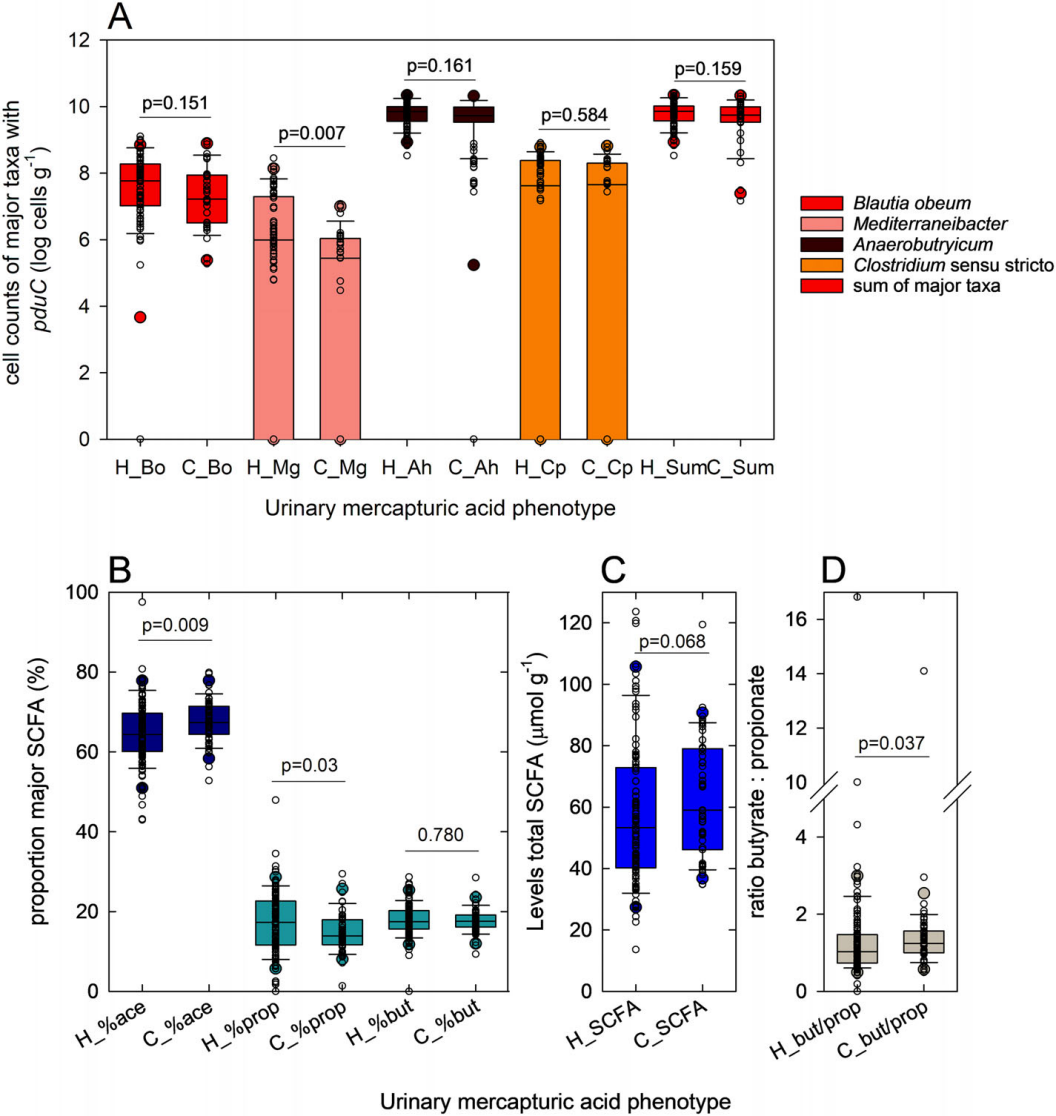
Abundance of *pduC*‐harboring taxa and fecal SCFA profiles in participants with CEMA or 3‐HPMA phenotype. Participants were separated into CEMA (C) or 3‐HPMA (H) phenotypes based on the predominant acrolein biomarker in feces. We reanalyzed *pduC* profiles determined with qPCR and investigated differences in fecal levels, proportions, and ratios of major SCFA acetate, propionate, and butyrate determined with HPLC‐RI. (A) Abundance of *pduC*‐harboring taxa in C and H phenotypes. (B) proportions of acetate (%ace), propionate (%prop), and butyrate (%but) of total SCFA (acetate + propionate + butyrate) in C and H phenotypes. (C) total SCFA levels in C and H phenotypes; (D) ratio of butyrate to propionate in C and H phenotypes. Statistical differences were determined with the Mann–Whitney *U* test, *p* < 0.05 was considered significant, and *p* < 0.1 a trend. (A)–(D) Shown are boxplots with the median and 25th and 75th percentiles; whiskers indicate the 5th and 95th percentiles, and each individual sample is presented as a white circle.

To also investigate whether such a phenotype is related to overall microbiota composition and the occurrence of taxa with *pduC* and fecal SCCA profiles, we separated the cohort based on urinary “CEMA” or “3‐HPMA” phenotypes and reanalyzed 16S rRNA gene sequences. There was a significant difference in β‐diversity of fecal microbiota in participants with different phenotypes (PERMANOVA *p* < 0.001, Figure S6). Based on LDA analysis, there were differences in abundance of taxa related to lactate metabolism [53], with a predominance of *Coprococcus* and *Bilophila* in participants with the “CEMA” phenotype and *Anaerostipes* with “3‐HPMA” (Table 1). *Coprococcus* spp. uses the acrylate pathway to metabolize lactate to propionate, *Desulfovibrionaceae* such as *Bilophila* use lactate as a cofactor during dissimilatory reduction of sulfate, and *Anaerostipes* forms butyrate from lactate [53]. The “3‐HPMA” group had significantly higher fecal levels of *M. gnavus;* abundance was also higher of *B. obeum* and *A. hallii/soehngenii*, albeit not significantly (Figure 7A). While participants with the “CEMA” phenotype had higher proportions of acetate, the “3‐HPMA” phenotype was characterized by significantly higher proportion of propionate (Figure 7B), a trend (*p* < 0.1) for lower total SCFA (sum of acetate, propionate, and butyrate) (Figure 7C), and a significantly lower ratio of the levels of butyrate to propionate (Figure 7D). These data suggest that while there was no difference in the taxonomic profiles based on β‐diversity, there was a relationship between mercapturic acid phenotypes, occurrence of specific functional groups, and fecal SCFA profiles.

**TABLE 1:** Difference in abundance based on phenotype.

Taxonomic level (phylum and lower taxonomic level)		Taxon	LDA score	Phenotype
*Bacillota*	Genus	*Allisonella*	2.1 ± 0.1	C
	Genus	*Catenibacterium*	2.8 ± 0.1	C
	Genus	*Agathobacter*	3.3 ± 0.1	C
	Family	*[Eubacterium] coprostanoligenes *group	3.7 ± 0.1	C
	Genus	un_f *[Eubacterium] coprostanoligenes* group	3.7 ± 0.1	C
	Family	*Erysipelotrichaceae*	3.2 ± 0.1	C
	Genus	*Holdemanella*	3.0 ± 0.1	C
	Genus	*Lachnospiraceae UCG‐003*	2.0 ± 0.1	C
	Genus	*Coprococcus*	3.7 ± 0.1	C
	Genus	*NK4A214 *group	2.7 ± 0.1	C
	Genus	*[Eubacterium] siraeum *group	2.8 ± 0.1	C
	Order	*Christensenellales*	3.8 ± 0.1	C
	Family	*Christensenellaceae*	3.8 ± 0.1	C
	Genus	*Christensenellaceae R‐7 *group	3.8 ± 0.1	C
	Genus	*Family XIII AD3011 *group	2.8 ± 0.1	C
	Genus	*Colidextribacter*	2.8 ± 0.2	C
	Family	*Anaerovoracaceae*	2.9 ± 0.1	C
	Order	un_c *Clostridia*	2.8 ± 0.1	C
	Family	un_c *Clostridia*	2.8 ± 0.1	C
	Genus	un_c *Clostridia*	2.8 ± 0.1	C
	Family	*Oscillospiraceae*	3.3 ± 0.1	C
	Genus	*Ruminococcus*	3.8 ± 0.1	C
	Family	*UCG‐010*	3.0 ± 0.2	C
	Genus	un_f *UCG‐010*	3.0 ± 0.2	C
	Genus	*Lachnospiraceae UCG‐004*	2.3 ± 0.1	C
*Thermodesulfobacteriota*				
	Class	*Desulfovibrionia*	2.1 ± 0.1	C
	Order	*Desulfovibrionales*	2.1 ± 0.1	C
	Family	*Desulfovibrionaceae*	2.1 ± 0.1	C
	Genus	*Bilophila*	2.1 ± 0.1	C
*Actinomycetota*	Genus	*CHKCI002*	2.0 ± 0.1	C
	Genus	*Slackia*	2.3 ± 0.1	C
*Bacillota*	Order	*Lactobacillales*	3.6 ± 0.1	H
	Genus	*Anaerostipes*	3.9 ± 0.1	H
*Actinomycetota*	Order	un_c *Coriobacteriia*	2.1 ± 0.1	H
	Family	un_c *Coriobacteriia*	2.1 ± 0.1	H
	Genus	un_c *Coriobacteriia*	2.1 ± 0.1	H
	Genus	*Senegalimassilia*	2.6 ± 0.0	H

*Note*: We classified fecal microbiota based on mercapturic acid phenotypes (C, CEMA, and H, 3‐HPMA) and performed linear discriminant analysis (LDA). LDA scores for taxa with an LDA > 2 and *p* < 0.01 are shown.

### 3.8 Concluding Remarks

Taken together, our data show that the potential for Pdu activity is a common function of the human gut microbiota and that the reliable presence of glycerol‐utilizing taxa allows for immediate transformation of dietary glycerol. The observations suggest that there is consistent potential for intestinal acrolein release. Fat/glycerol intake with the diet together with intestinal microbiota activity might temporarily increase endogenous acrolein formation; however, overall levels of intestinal acrolein were estimated as low. Based on the urinary predominance of biomarkers of acrolein, donors could be separated into 3‐HPMA or CEMA phenotypes and these mercapturic acid phenotypes were also linked to overall microbiota composition, the presence of utilizers of the fermentation intermediate lactate, and fermentation activity linked to *pduC*, suggesting a link between intestinal acrolein and the gut/kidney axis.

## Conflicts of Interest

The authors declare no conflicts of interest.

## Supporting information




**Supporting Information file 1**: mnfr70289‐sup‐0001‐SuppMat.docx

## Data Availability

The data that support the findings of this study are openly available in ENA at https://www.ebi.ac.uk/ena/browser/home, reference number PRJEB90537.
