# Supplementary material for: Microbial Transformation of Dietary Glycerol Contributes to Intestinal Acrolein Formation and Urinary Excretion
Source: Mol Nutr Food Res. 2025 Oct 29;69(24):e70289. doi: 10.1002/mnfr.70289 (PMC12700045; doi:10.1002/mnfr.70289)
Supplement: Supplementary file 1 — Supporting Information file 1: mnfr70289‐sup‐0001‐SuppMat.docx [file MNFR-69-e70289-s001.docx]

**Supplementary data for:**

**Gut microbial transformation of dietary glycerol contributes to intestinal acrolein formation and urinary excretion**

Clarissa Schwab^1^*, Hanna Lang^2^, Simone Stegmüller^2^, Jiri Hosek^1^, Angeliki Marietou^1^, Lucía Huertas-Díaz^1^, Qing Li^1#^, Asta Petrine Smedgaard Krings^1^, Andrea Zander^2^, Ulrik Kræmer Sundekilde^3^, Elke Richling^2^*

^1^Department of Biological and Chemical Engineering, Aarhus University, Aarhus, Denmark

^2^Department of Chemistry, Division of Food Chemistry and Toxicology, University of Kaiserslautern-Landau (RPTU), Kaiserslautern, Germany

^3^Department of Food Science, Aarhus University, Aarhus, Denmark

^#^current affiliation, Technical University of Denmark, Copenhagen, Denmark

*correspondence to:

Clarissa Schwab, Gustav Wieds Vej 10, 8000 Aarhus, Denmark, [schwab@bce.au.dk](mailto:schwab@bce.au.dk)

Elke Richling, Erwin-Schrödinger-Strasse 52/54, 67663 Kaiserslautern, Germany, elke.richling@chem.rtpu.de

**
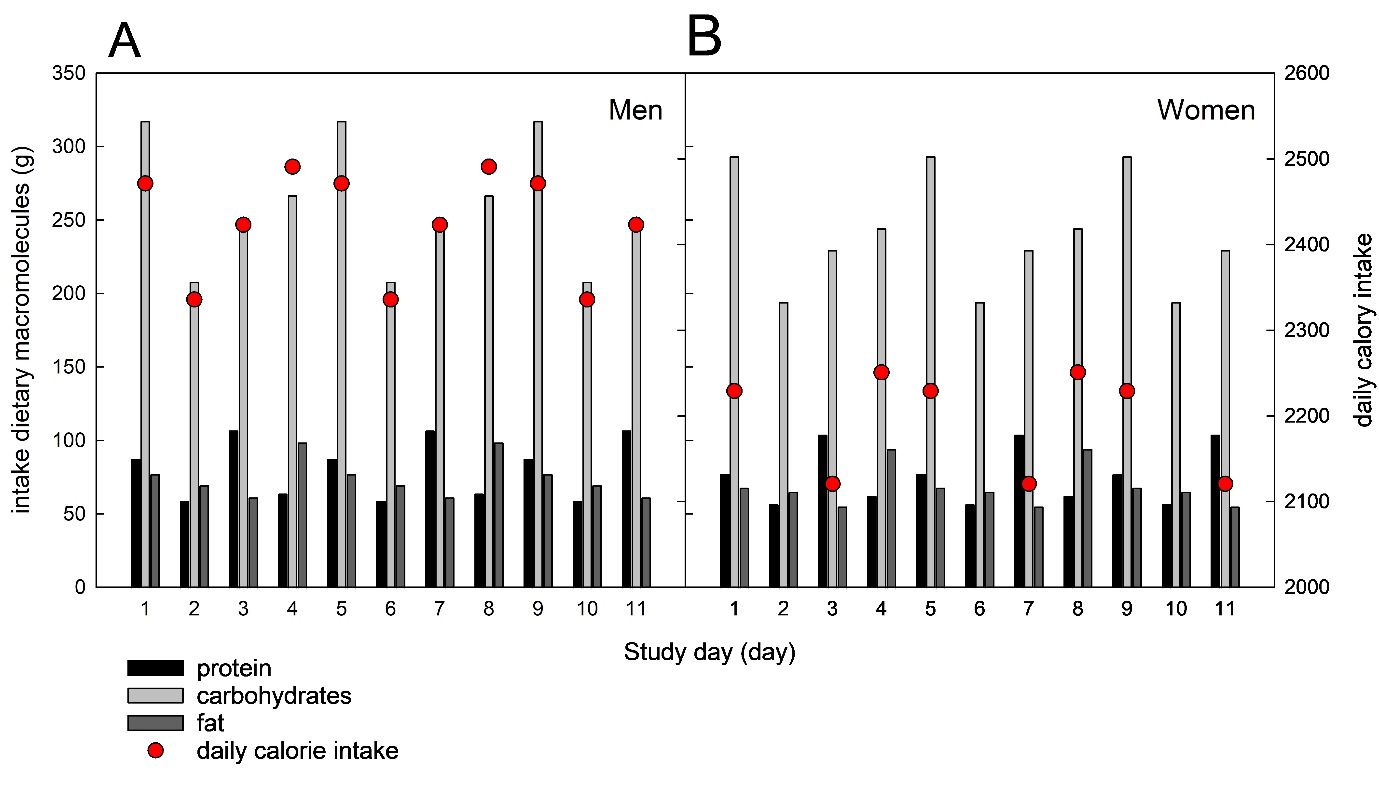
**

**Suppl. Fig. S1. Diet composition and daily calory intake.** Content of major biomolecules (protein, carbohydrates and fat) and daily calory intake provided with diets designed for male (A) and female (B) participants.


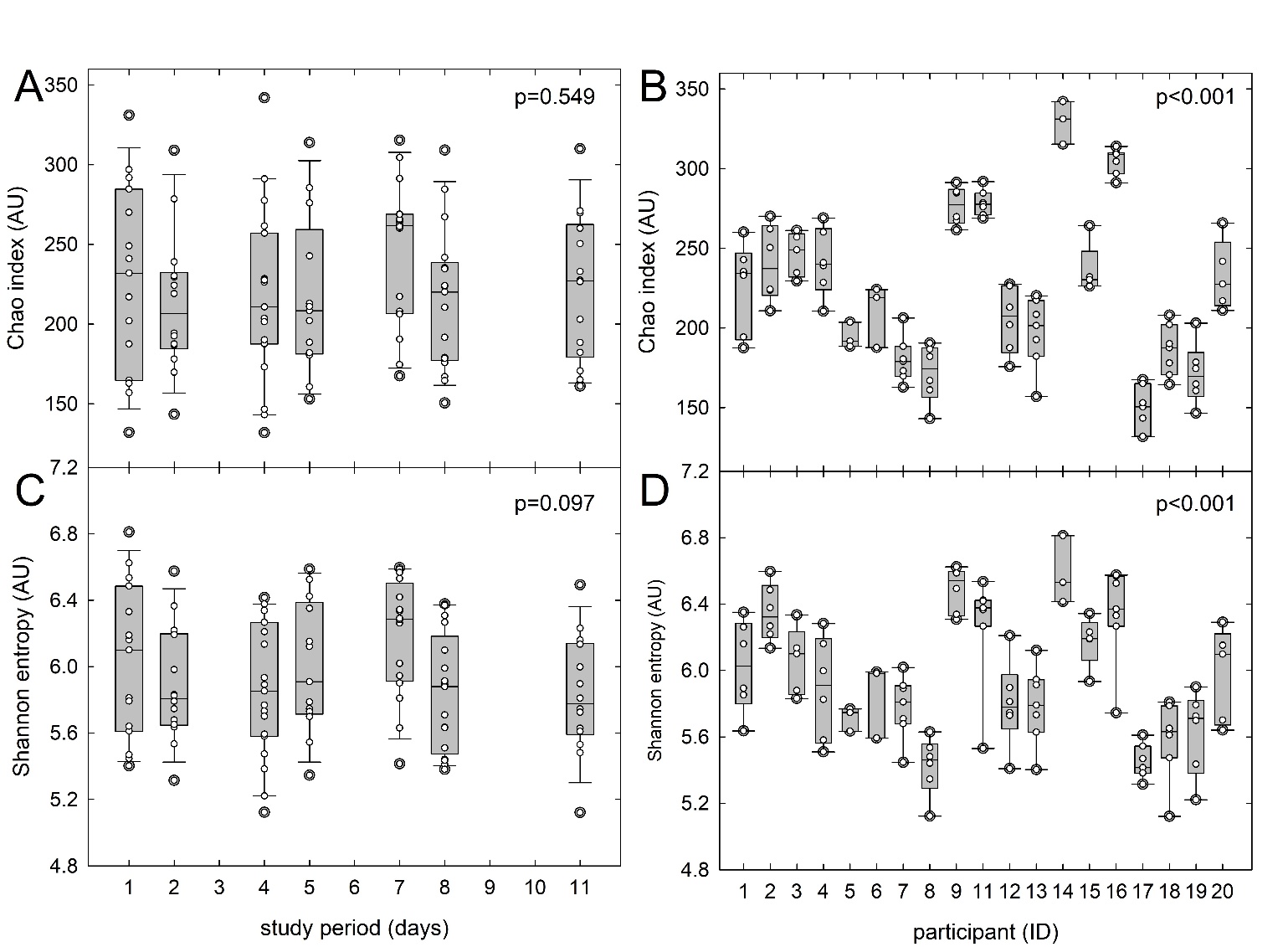


**Suppl. Fig. S2. Alpha diversity indices clusters by study days and donors.** Indices were calculated from 16S rRNA gene amplicon sequencing using QIIME2 [1]. (**A**) Chao index of fecal microbiota categorized in study days, (**B**) Chao index of fecal microbiota of each participant, (**C**) Shannon index of fecal microbiota categorized in study days, (**D**) Shannon index of fecal microbiota of each participant.


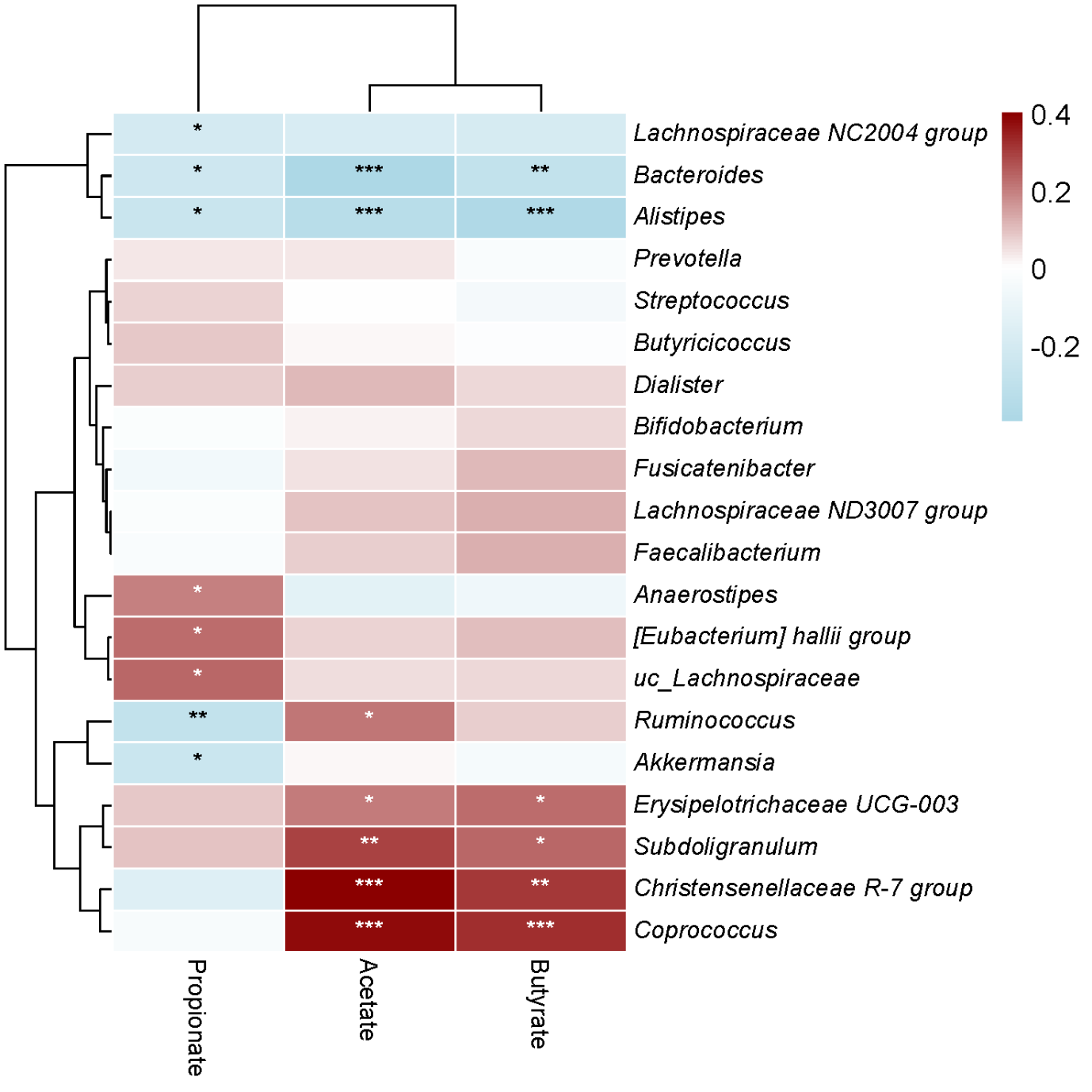
**Suppl. Fig 3. Relationship of relative abundance and fecal SCFA.** Heatmap showing Spearman correlation coefficients between relative abundance of the 20 most abundant genera (determined by 16S rRNA gene sequencing) and fecal SCFA levels (determined by HPLC-RI). Red indicates a positive correlation; blue indicates a negative correlation. Asterisks denote significance levels (*p* < 0.05, **p* < 0.01, ***p* < 0.001). *Eubacterium hallii* has been reclassified *Anaerobutyricium.*


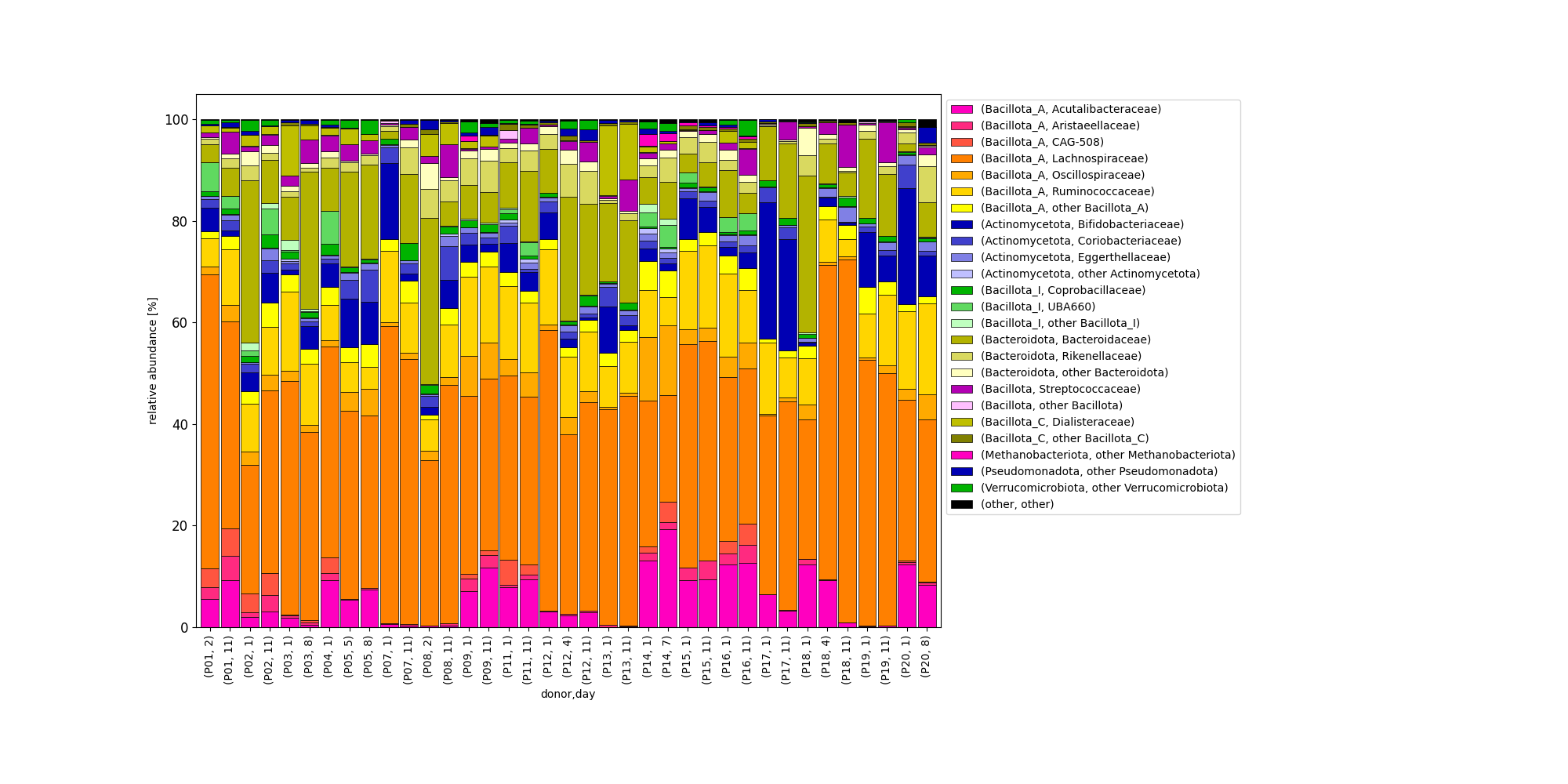


**Suppl. Fig 4.** **Metagenome taxonomic profiling based on family level**. We used sylph [2], which employs a k-mer containment method to taxonomically profile the metagenomes. P, donor ID; number, collection day of fecal sample. Average relative abundance of major phyla across all samples was: *Actinomycetota*: 9.21%, *Bacillota*: 2.43%, *Bacillota*_A 65.87%, *Bacillota*_C: 2.19%, *Bacillota*_I:  2.53%, *Bacteroidota*: 16.12%, *Cyanobacteriota*: 0.10%, *Methanobacteriota:* 0.18%, *Pseudomonadota*: 0.57%, *Verrucomicrobiota*: 0.68%, and other: 0.12%.


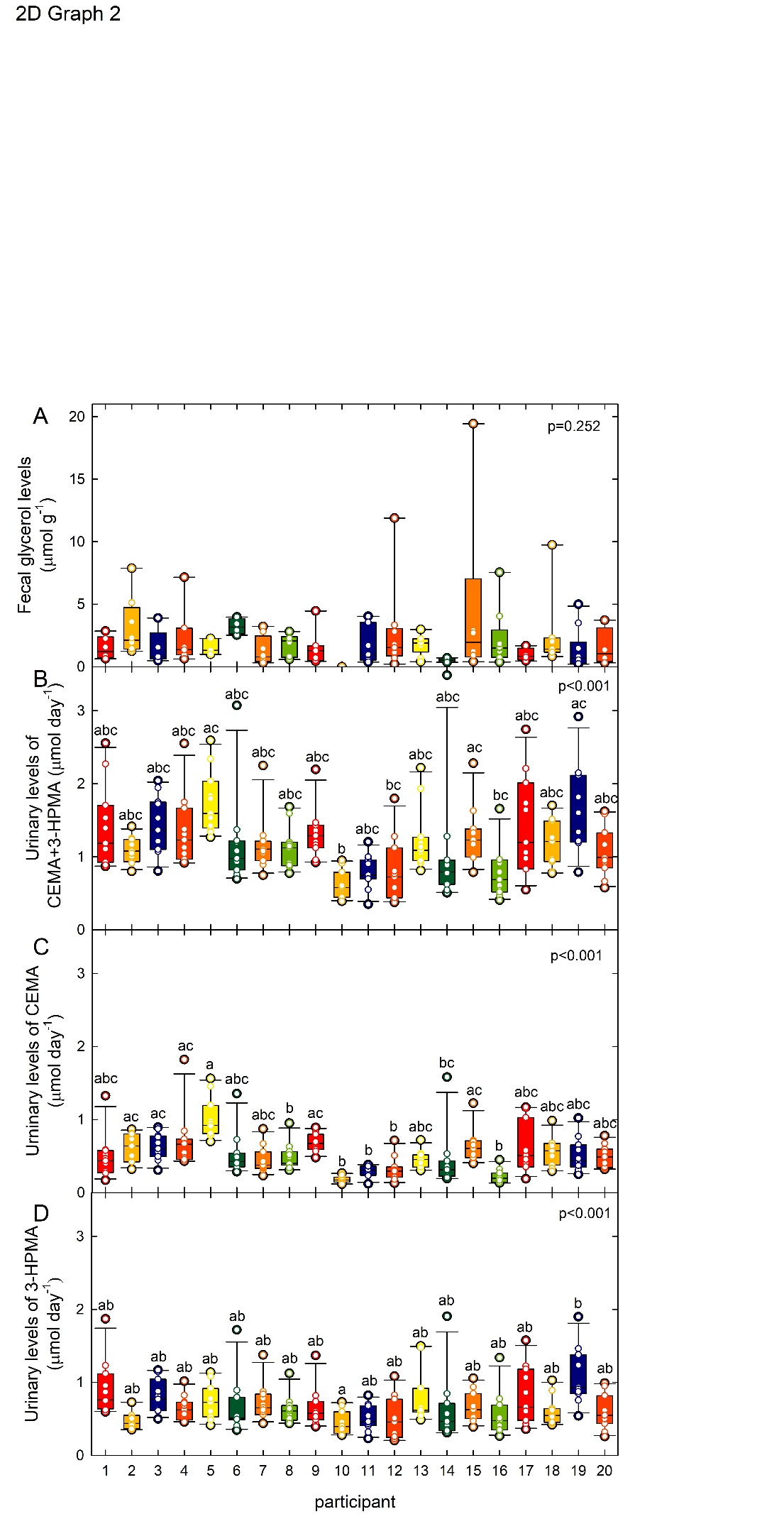


**Figure S5.** **Fecal and urinary biomarkers of glycerol metabolism of individual participants.** Fecal levels of glycerol were determined using ^1^H-NMR, while CEMA and 3-HPMA were detected with LC-MS. (**A**) Glycerol levels, (**B**) the sum of CEMA+3-HPMA, (**C**) CEMA levels, (**D**) 3-HPMA levels. Statistical difference was determined using Kruskal Wallis test with All Pairwise Multiple Comparison Procedures (Tukey Test), p < 0.05 was considered significant. Different small letters indicate significant difference of medians between samples of individual donors.

**
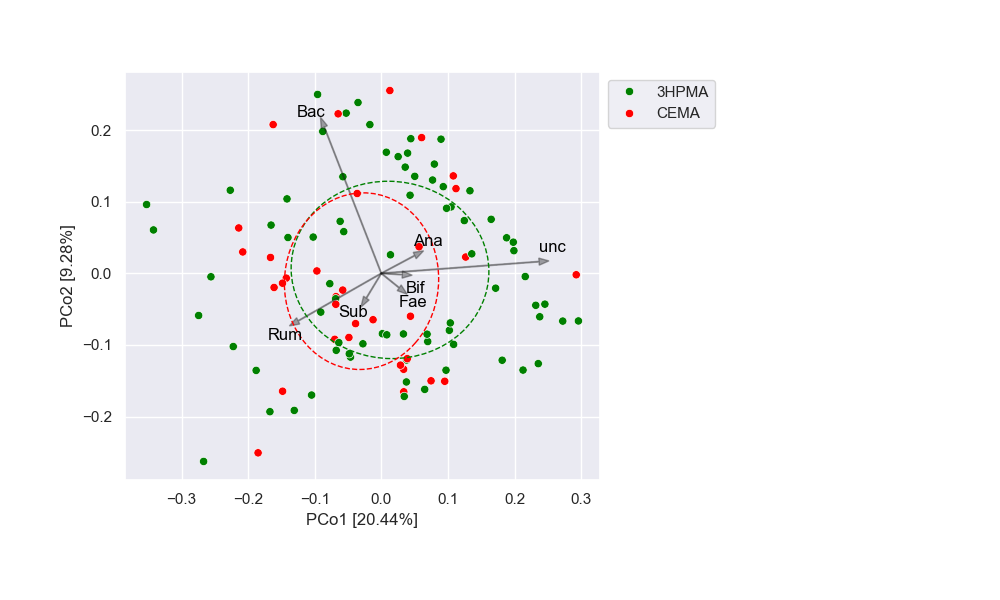
**

**Figure S6. Beta-diversity biplot of donor microbiota based on phenotype.** We separated donors based on predominant ‘CEMA’ and ‘3-HPMA’ phenotypes and determined beta-diversity based on Bray Curtis. Arrows indicate the genera that most contribute to localization of individual samples in the plot. Ana, *Anaerostipes;* Bac, *Bacteroides;* Bif, *Bifidobacterium;* Fae, *Faecalibacterium;* Rum,  *Ruminococcus;*  Sub , *Subdoligranulum*; unc, unclassified *Lachnospiraceae.*

**Suppl. Table S1.** **Food plan of the study.** Participants received four repetitive diet schemes, composition of the individual diet schemes and the days they were provided are shown

|  | **Dietary schemes 1-4** | | | |
| --- | --- | --- | --- | --- |
|  | **1** | **2** | **3** | **4** |
| days | 1, 5, 9 | 2, 6, 10 | 3, 7, 11 | 4, 8 |
| serving time | **Meal content** | | | |
| 8 h | Yoghurt, natural 3.5% | Yoghurt, natural 3.5% | Yoghurt, natural 3.5% | Yoghurt, natural 3.5% |
|  | Fruit salad: Apple, pear, banana, | Fruit salad: Apple, pear, banana, | Fruit salad: Apple, banana, grapes | Fruit salad: Apple, pear, banana |
|  | Oat flakes | Sunflower seeds | Oat flakes |  |
|  | Orange juice | Apple juice | Apple juice | Orange juice |
|  |  |  |  | Coffee 250 mL (day 4)/500 mL (day 8)^a^ |
| 12 h | Minced meatballs | Chicken fricassee | Shepherd's cheese | Herb butter |
|  | Pasta | Rice | Bulgur | Boiled potatoes |
|  | Tomato sauce | Butter | Kaiser vegetables | Kaiser vegetables |
|  | Apple juice | Orange juice | Orange juice | Apple juice |
| 14 h | Fruit (banana; orange) | Fruit (banana; apple) | Fruit (banana; orange) | Fruit (banana; orange) |
|  | Greek yoghurt 10% fat | Greek yoghurt 10% fat | Greek yoghurt 10% fat | Greek yoghurt 10% fat |
| 18 h | Boiled potatoes | Pasta (gnocchi) | Pasta (spaghetti) | Rice |
|  | Herb quark 40% fat | Tomato sauce | Carbonara sauce with cream and ham | Chicken fricassee |
|  | Yoghurt, natural 3.5% |  | Cooked turkey breast | Vanilla pudding |
|  | Apple juice | Apple juice | Apple juice | Apple juice |

^a^coffee was consumed as part of the original study design [3].

**Table S2**. **Primers used to quantify *pduC* of selected taxa using qPCR [4,5].**

| Target species | Primer sequences (5’-3’) | Reference |
| --- | --- | --- |
| *Anaerobutyricum hallii* and *Anaerobutyricum soehngenii* | F: CGTTATGCTCCATTTAATGCT  R: CCAAGGAGTATCATCACCATC | 4 |
| *Limosilactobacillus reuteri* | F: CGTTATGCACCATTCAATGCT  R: CCATGGAGTATCATCACCATC | 4 |
| *Ruminococcus gnavus* | F: CTGAAGGTCCGCTTTACATC  R: CAAACATATTGTCATAGTTCG | 4 |
| *Veillonella dispar* | F: CGTTATGCACCACTTAATGCG  R: CCAAGGTGTATCATCACCATC | 4 |
| *Blautia obeum* | F: CTGAAGGTACGTTTTACCTC  R: CGAACATATTGTCGTAGTTTG | 4 |
| *Clostridium* senso stricto | F: GTR GTT GAA ATG ATG ATG  R: AWG GWG TAT CRT CKC CAT | 5 |
| *Flavonifractor plautii* | F: CTGAAGATGCGCTTTACCTC  R: CGAACTACGACAACATGTTCG | 4 |
|  |  |  |

**Table S3**. **Classification scheme used to identify participants with CEMA or 3-HPMA phenotype**. We separated participants into urinary mercapturonic acid phenotypes based on how many days during the 11 day study the amount of excreted 3-HPMA was higher than CEMA, the differences in median levels of 3-HPMA and CEMA collected from the same donor, and the fold difference of the medians of 3-HPMA/CEMA for each participant. (**A**) Participants assigned to CEMA phenotype. A participant was assigned to the CEMA phenotype if urinary daily 3-HPMA levels were lower than CEMA at less than six out of 11 days, or if the absolute and fold difference of median 3-HPMA and CEMA levels was negative or < 1, respectively (indicated with shading). (**B**) Participants assigned to 3-HPMA. All participants assigned to the 3-HPMA phenotype had higher urinary daily 3-HPMA levels than CEMA at seven or more out of 11 days, absolute and fold difference of median 3-HPMA and CEMA levels was positive or >1, respectively.

**A**

| **Participants with CEMA phenotype** | | | | | | |
| --- | --- | --- | --- | --- | --- | --- |
| **Participant ID** | **2** | **4** | **5** | **9** | **15** | **18** |
| Days daily levesl 3-HPMA>CEMA | 3 | 7 | 2 | 3 | 5 | 6 |
| Difference in median levels of 3-HPMA -CEMA (µmol day^-1^) | -0.20 | -0.04 | -0.19 | -0.10 | 0.03 | -0.03 |
| Fold difference of median levels of 3-HPMA/CEMA | 0.69 | 0.94 | 0.79 | 0.86 | 1.04 | 0.95 |

**B**

| **Participants with 3-HPMA phenotype** | | | | | | | | | | | | | | |
| --- | --- | --- | --- | --- | --- | --- | --- | --- | --- | --- | --- | --- | --- | --- |
| **Participant ID** | **1** | **3** | **6** | **7** | **8** | **10** | **11** | **12** | **13** | **14** | **16** | **17** | **19** | **20** |
| Days daily levels 3-HPMA >CEMA | 10 | 11 | 8 | 9 | 9 | 11 | 11 | 9 | 11 | 9 | 11 | 10 | 11 | 7 |
| Difference in median levels of 3-HPMA - CEMA (µmol day^-1^) | 0.33 | 0.17 | 0.08 | 0.28 | 0.21 | 0.22 | 0.16 | 0.16 | 0.17 | 0.15 | 0.28 | 0.15 | 0.50 | 0.06 |
| Fold difference of median levels of 3-HPMA/CEMA | 1.75 | 1.28 | 1.20 | 1.74 | 1.51 | 2.29 | 1.52 | 1.56 | 1.37 | 1.48 | 2.45 | 1.30 | 2.13 | 1.23 |

**References**

1. E. Bolyen, J. R. Rideout, M. R. Dillon, N. A. Bokulich, C. C. Abnet, G. A. Al-Ghalith, *et al., Nat. Biotechnol.* **2019**, *37*, 852-857.
2. J. Shaw, Y. W. Yu, *Nat. Biotechnol****.* 2024,** https://doi.org/10.1038/s41587-024-02412-y.
3. D. Bohlen, D. Karlstetter, J. Leidner, J. I. Kremer, V. Kirsch, G. Eisenbrand, T. Bakuradze, S. Stegmüller, E. Richling, *Food Chem. Toxicol*. **2024**, *189*, 114774.
4. A. Ramirez Garcia, J. Zhang, A. Greppi, F. Constancias, E. Wortmann, M. Wandres, K. Hurley, A. Pascual-García, H. J. Ruscheweyh, S. J. Sturla, C. Lacroix, C. Schwab, *Environ Microbiol*. **2021**, *23*, 1765
5. Q. Li, H.-J. Ruscheweyh, L. H. Østergaard, M. Libertella, K. S. Simonsen, S. Sunagawa, A. Scoma, C. Schwab, *Microbiome.* **2024**, *12*, 178.
